# EP24 Arthritis mutilans coexisting with three separate granulomatous diseases

**DOI:** 10.1093/rap/rkaa052.023

**Published:** 2020-11-03

**Authors:** Ritsuko Saito, Ernest Wong

**Affiliations:** Queen Alexandra Hospital, Portsmouth, United Kingdom

## Abstract

**Case report - Introduction:**

Granulomatous disorders are diverse in their aetiologies and presentations. We present an unusual case of severe psoriatic arthritis patient who subsequently developed multiple granulomatous diseases over time, granulomatous interstitial nephritis, granulomatous sarcoidosis with hilar lymphadenopathy and localised laryngeal granulomatous inflammation secondary to lambda type amyloidosis.

**Case report - Case description:**

52-year-old gentleman with arthritis mutilans secondary to severe poorly controlled psoriatic arthritis was followed up in Rheumatology clinic. Earlier therapy with leflunomide and methotrexate provided inadequate control. Golimumab, despite giving a good response, was stopped in 2013 after 5 months of treatment due to acute kidney injury. Renal biopsy revealed granulomatous interstitial nephritis, thought to be Golimumab-induced based on the timing of usage and reversibility with discontinuation. He was then trialled on Ustekinumab and Secukinumab in 2016 and 2017 respectively with variable response.

He also had a few-years history of voice change (high pitched) and sore throat which he attributed to recurrent colds. He denied dysphagia or breathlessness, and he did not have stridor. He has never smoked and only drank alcohol occasionally. ENT team noted white deposits on erythematous and thickened false vocal cords and posterior glottis with a thin web on microlaryngoscopy, which histologically proved to show granulomatous inflammation, potentially consisting of amyloid, although congo red stain was negative. On further investigation, including SAP scan, he was diagnosed with localised lambda type amyloid. Increasing throat pain and worsening dysphonia prompted change of management from conservative to a surgery at a specialist centre and an input from speech and language therapy team.

During this time, consideration for Etanercept for his joint and skin disease was put on hold, pending further management of laryngeal amyloidosis. Furthermore, he presented to hospital with breathlessness in 2019, where his chest X-ray showed bulky right hilum and a follow-up CT chest revealed calcified right hilar and mediastinal lymphadenopathy, ground glass opacification and consolidation. Histology from hilar node was suggestive of sarcoidosis, with stain negative for amyloid.

He underwent removal of false vocal cords for his symptomatic laryngeal amyloidosis. He continues to be followed up at the local Rheumatology, Dermatology and ENT team.

**Case report - Discussion:**

Granulomatous diseases have vast aetiologies, including infectious, immunological, neoplastic, and chemical-induced processes. The age at which they affect patients and tissue they involve also vary hugely. This is the first reported case of three seemingly unrelated granulomatous diseases occurring in a single patient with severe refractory psoriatic arthritis. Retrospective reassessment of the histology samples supported that these are three separate pathologies. It is very unusual for one patient to acquire multiple separate granulomatous diseases, which was why the diagnostic process of this patient was challenging. In this case, managing the original underlying psoriatic arthritis was particularly difficult due to interruptions of treatment for adverse drug effects and investigations and treatment of subsequent granulomatous diseases. The case also raises questions about possible currently unknown association between the pathologies.

**Case report - Key learning points:**

Key points are the uniqueness of this case and that it highlighted the possibility of currently under-reported association between these three granulomatous conditions. As ever, a multidisciplinary approach to managing such a complex patient is important for the provision of good care.

